# Evaluation of Psychosocial Pathways to Family Adaptation of Chinese Patients With Liver Cancer Using the McCubbin's Family Resilience Model

**DOI:** 10.3389/fpsyt.2021.703137

**Published:** 2021-12-16

**Authors:** Shirou Mao, Huijuan Lu, Yuxia Zhang, Jingxian Yu, Xiaorong Li, Jian Peng, Yan Liang

**Affiliations:** ^1^School of Nursing, Fudan University, Shanghai, China; ^2^Zhongshan Hospital, Fudan University, Shanghai, China

**Keywords:** family resilience, family adaptation, liver cancer, model, influencing factors

## Abstract

**Background and Aim:** With the prolonged survival time of patients with liver cancer, these families may face tremendous pressure and development dilemmas that can easily lead to family adaptation crises. Correspondingly, family adaptation crises adversely affect the quality of life of patients and family members. Basing on McCubbin's resilience model of family stress, adjustment, and adaptation, and considering the key factors affecting family resilience based on a review of literature, this study involved a construction of a family adaptation influencing factors model in Chinese liver cancer patients, which was then verified and revised.

**Methods:** This cross-sectional study was conducted between August and December 2020. Using convenience sampling, we selected 265 liver cancer families from the liver tumor center of a teaching hospital affiliated with a university in Shanghai, China. Data from 252 patients with liver cancer and their caregivers were used to identify the factors and pathways associated with family adaptation. The relationships were modeled using structural equations.

**Results:** A total of 265 liver cancer families participated in the survey, and 252 valid questionnaires were returned, with a response rate of 95.09%. The pathway regression coefficients of six factors (family burden, individual resilience, family problem-solving and coping, inner family support, outer family social support, and family function) in the model were found to be statistically significant (*P* < 0.05), indicating that all of them were significantly associated with family adaptation. Among them, inner family support, outer family social support, and family function were direct influencing factors, while the others were indirect. The path coefficients of the total effect of the determinants on family adaptation were as follows (from largest to smallest): individual resilience (0.562), family function (0.483), outer family social support (0.345), family burden (−0.300), inner family support (0.293), family problem-solving and coping (0.127).

**Conclusions:** Our findings suggest that clinical nurses should not only pay particular attention to direct influencing factors, develop strategies to strengthen the overall family function, encourage patients and caregivers to utilize inner family and outer family social support, but should also consider indirect influence factors, focus on the vital role of the individual, and promote patients' and caregivers' personal and family coping ability.

## Introduction

According to global cancer data in 2020, primary liver cancer (henceforth referred to as liver cancer) is the sixth most common cancer and the third leading cause of cancer-related deaths worldwide, with approximately 906,000 new cases and 830,000 deaths in that year (1). Patients with liver cancer experience adverse symptoms and psychological burdens, low health-related quality of life, and high cost of treatment, which has become a major public health burden on a global scale (2, 3). China is one of the high-incidence areas, accounting for over 50% of new cases and deaths worldwide. Although liver cancer incidence and mortality rates have shown a decreasing trend, scholars estimated that the burden of liver cancer in China would still be severe by 2030 (4). With the growing sophistication of medical technology, the overall survival rate of liver cancer has increased, and survival time has been prolonged. As reported by Lencioni et al. (5), the survival rates of patients with liver cancer reached 70.3, 51.8, and 40.4% in the 1st, 2nd, and 3rd years after interventional therapy, respectively. In this situation, many families would be required to coexist with patients with liver cancer for a prolonged period.

Patients with liver cancer experience adverse physical symptoms and psychological problems in disease treatment and rehabilitation (6), and caregivers may experience anxiety and depression due to the influences of care burden, the uncertainty of the patient's disease progression, and development deprivation (7). At the same time, the diagnosis of liver cancer not only affects the individuals in the family, but also affects their relationship and family dynamics, which may lead to deterioration of the relationship between family members and changes in family lifestyles and values. Being a primary social group to maintain individual survival and development, the family is an important functional unit for achieving emotional communication and meeting the various development needs of family members. In the particular period, wherein cancer is confirmed, the family plays a powerful role and serves as the core force to help patients and family members deal with cancer. Families may face a severe crisis if they cannot effectively adapt to meeting the impact of liver cancer. Meanwhile, crises may further reduce their quality of life and life satisfaction (8). Therefore, the promotion of family adaptation to patients with liver cancer has become a problem lately.

Studies about liver cancer have mainly been conducted from a personal perspective, focusing on patients' symptoms, their negative psychological reactions, or caregiver burden (6, 7, 9). In recent years, some researchers have begun to explore the key role of family function in the treatment and recovery of cancer patients from the family's perspective as a whole (10). Upon analyzing the scientific literature, it appears that previous studies on families of patients with liver cancer mostly pay attention to the negative aspects of family experience and often ignore its internal advantages and positive factors. With the development of positive psychology, many researchers in medicine have shifted their focus from problems *per se* to the positive impacts produced by the family while coexisting with the patient, believing that the family has the potential to grow in adversity.

Family resilience is defined as individuals' and families' capacity to draw on mutual strengths to cope with or adapt to adversity using various resources in the interaction of multiple systems when encountering stressors. It is not a static structure but a process of positive interaction between individuals, families, and the external environment (11). An investigation on families of patients with stroke (12) revealed that family resilience is an essential factor that can positively and independently predict family adaptation. This conclusion has been verified in a study on families of patients with cancer (13), dementia (14), and children with illnesses (15, 16). Li et al. (17) studied the relationship among family resilience, individual resilience, and caregiver burden in breast cancer patients and found that family resilience and individual resilience can effectively alleviate the burden on caregivers and improve their quality of life. The study by Yan et al. (18) on families of patients with breast cancer also emphasized the importance of family resilience and concluded that intervention programs based on family resilience should be designed to enhance family adaptability and improve quality of life.

At present, a problem we must face is how to solve the plight of families with liver cancer patients and promote their family adaptation. The number of studies on family adaptation of liver cancer in China is few, and there is a lack of theoretical and systematic guidance. In this context, from the perspective of positive psychology, applying the theory of family resilience to the exploration of the family adaptation of patients with liver cancer may focus on exploring family advantages and support resources in disease treatment and rehabilitation. Additionally, it may explore new ways to enhance family adaptation, improve quality of life, and provide references and basses for research on families of patients with liver cancer.

### Conceptual Framework

This study used McCubbin's resilience model of family adjustment and adaptation to determine the multiple factors that affect family adaptation in patients with liver cancer. This model is developed from the ABC-X model (19), including two phases: the adjustment phase and the adaptation phase. The adjustment stage means that the family can achieve a good state through fine-tuning when facing mild or short-term stress. The adaptation stage means that if major stressful events lead to maladjustment, the family will change the way it operates. In this manner, family resilience to cope with the pressure is stimulated to regain balance and harmony. Family resilience is affected by family function, resources, cognition, problem-solving ability, and coping. This study uses the “adaptation stage” as the basic theoretical framework. The family burden, which is caused by liver cancer, would influence family adaptation by stimulating and adjusting the process of family resilience.

Mccubbin and Mccubbin (20) defined family resilience as the process by which individuals, families, and the external environment interact positively. Wu et al. (21) and Benzies and Mychasiuk (22) also redefined the protective factors of family resilience from three levels: individual, family, and social, affirming the important role of individuals as the basic functional unit of families. The stimulation and adjustment of family resilience is a process from the individual to family levels; however, there is no specific description of personal factors in McCubbin's model. Therefore, this study adds personal factors to the basic model. Resilience reflects positive beliefs of an individual or family when faced with adversity or crisis. It refers to an individual's ability to maintain and restore mental and physical health when faced with stress or adversity (23). It is an internal resource related to personality and can change the mindset of patients and their families in response to stressful events. Studies conducted by Yao and Qiu (24) and Chen et al. (25) suggest that enhancing personal resilience is an essential influencing factor in enhancing family resilience. Therefore, this study hypothesized that individual resilience is an important intermediary factor between family burden and family outcomes. Family burden affects the behavior of family members by affecting individual resilience and has an effect on family adaptation by influencing other factors.

At present, studies have confirmed the positive predictive effect of personal resilience on other family factors. Studies show that higher levels of individual resilience indicate better family function, higher levels of perceived support, and better family problem-solving and coping abilities (26–28). According to McCubbin's family resilience theory, other family factors work together to cope with the adverse effects of stressful events through interaction to promote the family to achieve an excellent adaptive state (11). Among them, family problem-solving and coping can affect support and resources as perceived by the family. Over time, it causes changes in family functions and the relationship between the family and the outside world (29). Zhang et al. (30) pointed out that social support is a key factor affecting family functioning. Meanwhile, the outer family social support system also affects the perception of inner family support system, which in turn affects family function and family adaptation (31).

**Figure 1:**
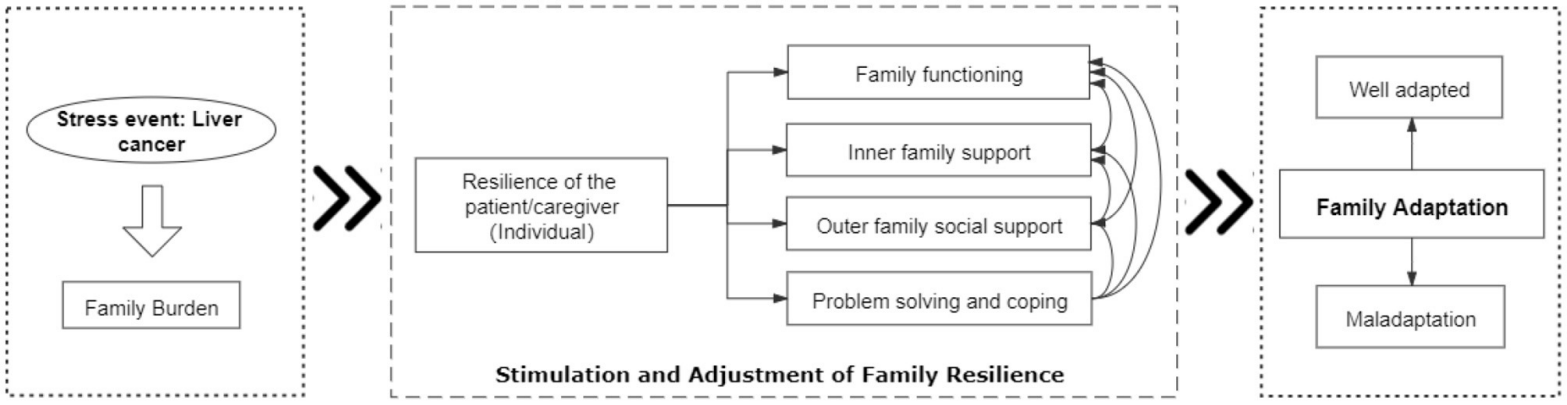
Model of influencing factors of family adaptation based on the Family Resilience Model—Conceptual framework.

Therefore, based on “McCubbin's resilience model of family adjustment and adaptation” and the literature review, this study proposed a model of influencing factors of family adaptation for patients with liver cancer (Figure 1). This study made assumptions about the relationship between variables based on the theoretical model: ① Family burden, caused by liver cancer, would affect individual resilience, which would, in turn, affect other family factors and ultimately affect family adaptation. ② Individual resilience of patients with liver cancer and caregivers would affect family adaptation by affecting family problem-solving and coping, outer family social support, inner family support, and family function. ③ Family problem-solving and coping, outer family social support, inner family support, and family function of liver cancer families interact and ultimately, directly and indirectly, affect the family adaptation of liver cancer families. ④ Family problem-solving and coping would affect outer family social support, inner family support, and family function. Outer family social support would affect inner family support and family function, and inner family support would affect family function.

Therefore, this study intends to comprehensively review the literature, construct and verify an influencing factor model of family adaptation of patients with liver cancer based on the family resilience theory, so as to provide new ideas and perspectives for improving the family adaptation of liver cancer families.

## Materials and Methods

### Participants and Procedure

The targeted population was the families of patients with primary liver cancer, including patients and their caregivers. Family members were recruited from the liver tumor center of a teaching hospital affiliated with a university in Shanghai, China. They were eligible to participate in this study if they fulfilled the following criteria: (a) a family member (≥18 years old) was diagnosed with primary liver cancer, (b) had a fixed primary family caregiver (≥18 years old), (c) was able to communicate in the language required for the study, and (d) volunteered to participate in this study. The exclusion criteria were as follows: (a) the caregiver was paid, and (b) the patient or caregiver had a history of psychiatric problems.

This study used the structural equation model for statistical analysis, and the sample size was calculated based on it. There is no precise formula for estimating the sample size required for the structural equation model analysis. Wu (32) reported that the sample size needed for structural equation model analysis is preferably >200. Therefore, the sample size of this study was estimated to be more than 200 cases.

After the approval of the ethics committee, the investigator collected the data using self-assessment questionnaires in the liver cancer wards. The questionnaires for patients included the general situation questionnaire and the Resilience Scale Specific to Cancer. The questionnaires for caregivers included a general situation questionnaire, Family Burden Scale of Disease, 10-item Conner-Davidson Resilience Scale, Family Crisis-Oriented Personal Evaluation Scales, Perceived Social Support Scale, Family APGAR (adaptation, partnership, growth, affection, resolve) Index, and Family Adaptation Scale. Fifteen families were pre-surveyed before the formal investigation. During the investigation, researchers selected eligible participants in accordance with the inclusion and exclusion criteria, and conducted on-site investigations using a unified protocol. Those who had difficulty writing due to educational level, eyesight, or other reasons were assisted by researchers to complete the questionnaire. A total of 265 pairs of liver cancer patients and their families participated in the survey, and 252 pairs of valid questionnaires were returned, with a response rate of 95.09%. Invalid questionnaires were defined as having missing data of more than 10% for one or more variables.

### Measures

#### Resilience Scale Specific to Cancer

A 10-item Resilience Scale Specific to Cancer, RS-SC-10 (33), was used in this study. RS-SC-10 contains 10 items with high discriminative parameters from the RS-SC and consists of two factors: Generic and Shift-Persist. The participants responded to the questionnaire using a 5-point Likert scale (from 1 = never to 5 = always), with the possible score range being 10–50. Higher scores indicate a higher level of resilience. The Cronbach's alpha coefficients were 0.85 (Generic) and 0.89 (Shift-Persist), respectively, based on the current participants.

#### 10-Item Conner-Davidson Resilience Scale

The 10-item Conner-Davidson Resilience Scale, CDRISE-10 (34, 35), consists of ten items rated on a 5-point Likert scale (from 1 = not at all true to 5 = strongly agree). It was developed as a brief version of the full 25-item CDRISE. The Chinese version was used in this study to measure caregivers' levels of resilience. The concurrent validity and internal consistency reliability of the Chinese version of the CDRISE-10 have been shown to be adequate (35). Based on this study, the internal consistency coefficient was 0.81.

#### Family Burden Scale of Disease

The Family Burden Scale of Disease, FBS (36), was used to assess family burden and stressors in six areas: family economic burden (six items), family daily activities (five items), family leisure and entertainment activities (four items), family relationships (five items), family members' physical health (two items), and family members' mental health (three items). The participants responded to the questionnaire using a 3-point Likert scale (from 0 = no burden to 2 = severe burden), and the score ranged from 0 to 50. A high score indicates a higher level of burden. Based on the current participants, the internal consistency coefficient of each dimension ranged from 0.69 to 0.7 (37).

#### Family Crisis-Oriented Personal Evaluation Scales

The Family Crisis-Oriented Personal Evaluation Scales, F-COPES (11), is a self-assessment scale used to measure the levels of family problem-solving and coping, and is completed by family members. Wang et al. (38) revised the scale into a Chinese version, which includes five dimensions: getting support (including support from family, relatives, friends, and neighbors), positive cognition, seeking support from spirit and belief, seeking social support (including support from other families, social institutions, doctors, and professionals), negative cognition, and avoidance. On this scale, participants were asked to report whether they agreed (from 1 = not at all true to 5 = strongly agree) to applying the family problem-solving and coping behaviors described for each item. The score range is 27–135. A higher score indicates a higher level of family problem-solving and coping. Based on the results of the current study, the internal consistency coefficient was 0.842.

#### Perceived Social Support Scale

The Perceived Social Support Scale, PSSS (39, 40), is a tool to measure self-perceived multi-level social support. There are 12 items in the scale, which can be divided into two dimensions: inner family support and outer family social support. The total score reflects the overall level of social support that individuals feel. The participants responded to the questionnaire using a 7-point Likert scale (from 1 = not at all true to 7 = strongly agree), with the possible score range being 12–84. A high score indicates a higher level of self-perceived social support. This scale is widely used worldwide, and has proven to be reliable and valid. Based on this study, the internal consistency coefficient was 0.88.

#### Family APGAR Index (APGAR)

The Family APGAR Index, APGAR (36), evaluates family function in five areas given as follows: adaptability, partnership, growth, emotion, and cohesion. The participants responded to the instrument using a 3-point Likert scale (0 = almost rarely, 1 = sometimes, 2 = usually). The scores are added together, with 0–3 points indicating severe family dysfunction, 4–6 points indicating moderate family dysfunction, and 7–10 points indicating good family function. Based on this study, the internal consistency coefficient was 0.813.

#### Family Adaptation Scale

The Family Adaptation Scale FAS (41), is used to assess the level of family adaptation of the disabled family, which is completed by family members. The scale was revised by Wang et al. (42) in Chinese and has been found to have satisfactory internal consistency reliability. The revised version consists of 15 items that describe satisfaction with family life using a 7-point Likert scale (from 1 = not at all to 7 = totally satisfied). The total score is the sum of all items. A higher score indicates a higher level of family adaptation. Based on this study, the internal consistency coefficient was 0.951.

### Data Analyses

A structural equation model was applied to confirm the hypothesis model using Amos version 24.0. Harman's single factor test method was used to test common method bias. Descriptive statistics were computed for the variables and reported as means, standard deviations, kurtosis, and skewness. Then, to build the best-fitted structural model, we proceeded step-by-step. First, a measurement model of family resilience was developed to assess family resilience. Second, the hypothesized model of family adaptation based on the family resilience model was developed. Then, the measured values were substituted into the model to perform structural equation model analysis to estimate the degree of fit between the hypothetical model and the actual data. The model was revised until the degree of fit met the standards. After validating the final model, the total effects of the factors (direct plus indirect *via* mediating relationships) were calculated from the standardized regression coefficients. The difference was statistically significant (*P* < 0.05).

To evaluate the model fit, a set of fit indices were used based on recommended criteria (32), including the following: the Chi-Square to df Ratio (χ^2^/df), when values between 1 and 2 indicate that the model fits well; a comparative fit index(CFI)≥0.90; goodness-of-fit index (GFI) ≥0.90; adjusted goodness-of-fit index (AGFI) ≥0.90; the Tucker–Lewis index (TLI) ≥0.90, which showed an acceptable fit of the model; the root mean square error of approximation (RMSEA), where values between 0.05 and 0.08 indicate that the model is acceptable, with <0.05 regarded as an appropriate fit; and the standardized root mean square residual (SRMR) of <0.05.

According to the literature review, the indirect effect value = (action path coefficient of the independent variables, which act on the first mediator variable on the indirect pathway) × (total effect on the family adaptation of the first mediator variable that was affected upon by the independent variable). The total indirect effect value is the sum of the indirect effect values of all paths from the independent variable to the dependent variable. For example, outer family social support (OFSS) acts on family adaptation (FA) indirectly through inner family support (IFS), and its indirect effect on FA should be the direct effect of OFSS to IFS multiplied by the total effect of IFS to FA. That is, the indirect effect value = 0.488^*^0.293 = 0.143.

## Results

### Demographic Characteristics

Mean ages of the family caregivers and cancer patients were 43.96 ± 11.87 and 56.33 ± 10.87 years, respectively. Other demographic and clinical information for caregivers and patients is shown in Table 1.

**Table 1:** Demographic and cancer-related characteristics.

**Variable**	**Liver cancer patients (*n* = 252)**	**Family caregivers (*n* = 252)**
	***N*** **(%)**	***N*** **(%)**
**Age (years)**		
≤ 40	24 (9.5)	106 (42.0)
41–64	172 (68.3)	134 (53.2)
≥65	56 (22.2)	12 (4.8)
**Gender**		
Male	222 (88.1)	78 (31.0)
Female	30 (11.9)	174 (69.0)
**Educational level**		
Junior high school or below	136 (54.0)	89 (35.2)
High school	66 (26.2)	78 (31.0)
University degree or above	50 (19.8)	85 (33.8)
**Time since diagnosis (months)**		
≤ 6	98 (38.9)	
7–12	27 (10.7)	
13–24	45 (17.9)	
≥25	82 (32.5)	
**Family type**		
Nuclear	109 (43.3)	
Stem	115 (45.6)	
Extended	28 (11.1)	
**Family income per month (CNY)**		
≤ 1,000	37 (14.7)	
1,000–2,999	64 (25.4)	
3,000–4,999	73 (29.0)	
≥5,000	78 (30.9)	
**Occupational status**		
Retired at home or Left work due to caregiving duties		114 (45.2)
Part-time/full-time job		138 (54.8)
**Relationship with patients**		
Spouses		177 (70.2)
Grown-up children		62 (24.6)
Others (parents/sisters/brothers)		13 (5.2)

### Common Method Bias Test Results

Results of the Harman's single factor test showed that the first common factor obtained without rotation explained 27.47% of the variance, which is less than the critical value of 40%. Therefore, we believe that there was no serious common method bias problem in this study.

### Descriptive Analysis

Descriptive statistics for observed variables were tested to check for normality of distribution. For each of the observed variables, the kurtosis and skewness values were between 1 and −1.2; therefore, this sample can be defined as having a normal distribution. The collinearity test in this study showed that the tolerance was >0.1, and the variance expansion factor (VIF) value was <10, indicating no serious collinearity problem. Descriptive statistics for the observed variables are presented in Table 2.

**Table 2:** Means, standard deviations, skewness, and kurtosis for observed variables.

**Variables**	**M**	**SD**	**Skewness**	**Kurtosis**
PR	35.96	7.226	−0.362	−0.359
CR	24.65	7.215	0.114	−0.428
OFSS	39.56	9.538	−0.447	0.117
IFS	22.06	4.732	−0.859	0.760
PSC	90.92	12.538	0.156	−0.152
FA	76.00	18.350	−0.890	0.695
Adaptability	1.52	0.561	−0.625	−0.652
Partnership	1.44	0.612	−0.610	−0.556
Growth	1.43	0.618	−0.595	−0.571
Emotion	1.51	0.582	−0.704	−0.480
Cohesion	1.60	0.587	−1.166	0.361
Economic burden	7.85	3.395	−0.476	−0.791
Daily Activity burden	3.86	2.492	0.253	−0.637
Leisure burden	3.56	2.288	0.081	−0.897

*PR, patient resilience; CR, caregiver resilience; OFSS, outer family social support; IFS, inner family support; PSC, problem-solving and coping; FA, family adaptation*.

### Measurement Model

Confirmatory factor analysis was used to verify whether “family resilience” included the core sub-concepts described above. The model was assessed using the maximum-likelihood method. Since this model was used to verify whether the concept of “family resilience” includes the corresponding sub-core concepts, the correlation between these sub-core concepts was not considered.

**Figure 2:**
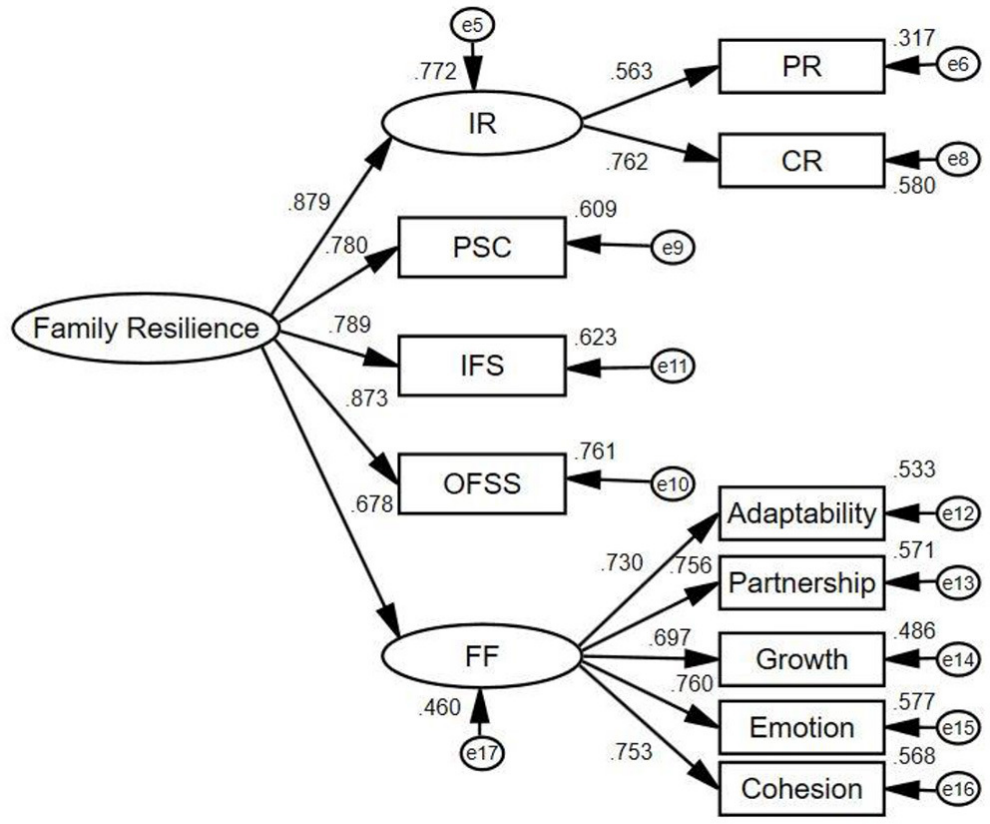
Measurement model used to calculate the latent variable—family resilience. IR, individual resilience; PR, patient resilience; CR, caregiver resilience; OFSS, outer family social support; IFS, inner family support; PSC, problem-solving and coping; FF, family function.

A test of the measurement model showed an acceptable fit to the data (χ^2^/df = 2.331, CFI = 0.963, GFI = 0.944, AGFI = 0.906, RMSEA = 0.073, SRMR = 0.0500), which supports the convergent validity of the indicators(Figure 2).

### Structural Model

**Figure 3:**
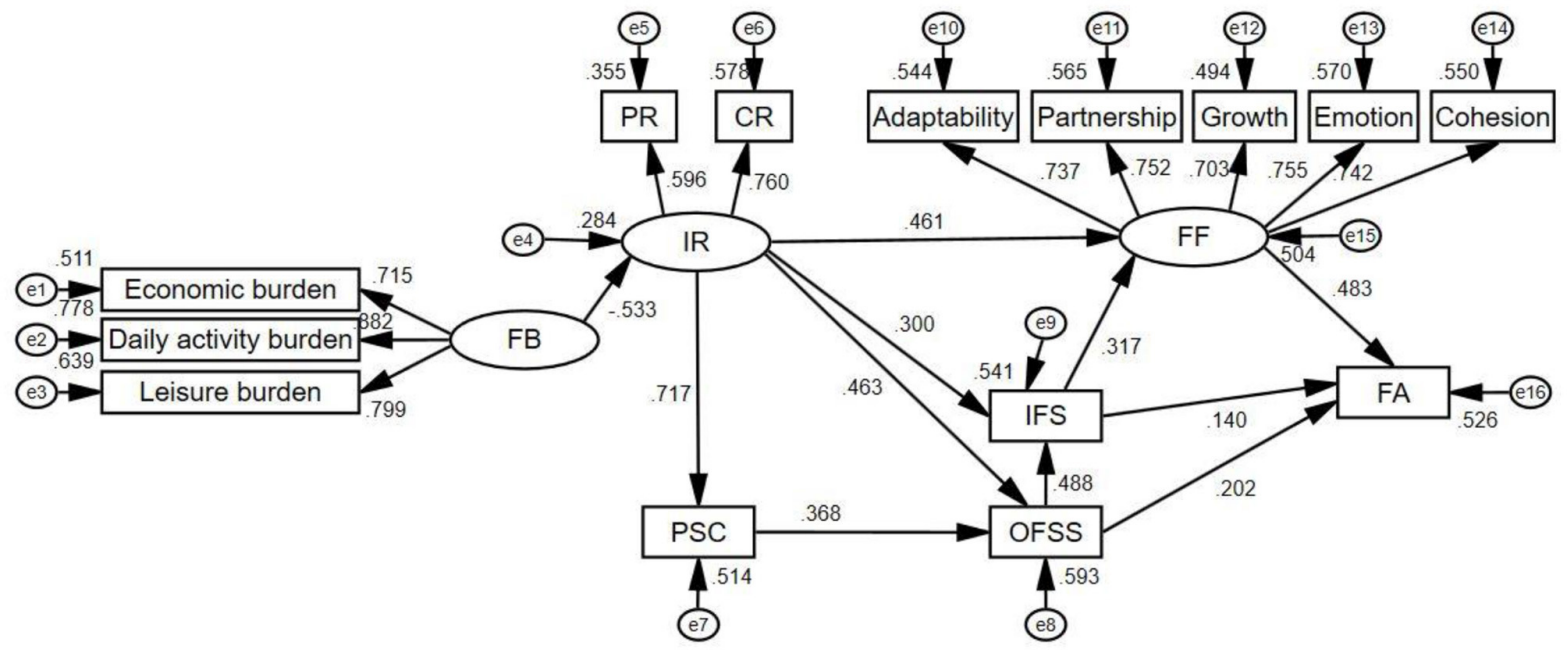
Model of influencing factors of family adaptation based on the Family Resilience Model—Final version. FB, family burden; IR, individual resilience; PR, patient resilience; CR, caregiver resilience; OFSS, outer family social support; IFS, inner family support; PSC, problem-solving and coping; FF, family function; FA, family adaptation.

This structural equation model analysis was applied to examine the effects of different factors on family adaptation and influential pathways. In the structural equation analysis, the hypothetical model was first tested, and included the possible paths among family burden, individual resilience, family problem-solving and coping, family function, inner family support, outer family social support, and family adaptation. Among them, four paths, including family problem-solving and coping to inner family support, family function, and family adaptation, as well as outer family social support to family function, are not significant. Based on previous literature and on our own findings, we deleted these paths. The final model is shown in Figure 3. The fit indices of the modified model were as follows: chi-square = 1.649, RMSEA = 0.051 [P(RMSEA) < 5%] = 0.573, SRMR = 0.043, CFI = 0.974, GFI = 0.939, and TLI = 0.966. Therefore, all paths were close to the ideal values, indicating that the modified model sufficiently fits the data.

Standardized direct effects, indirect effects, and total effects of standardization of all factors explaining the level of family adaptation are presented in Table 3, along with the path of effect generation. The results showed that the pathway regression coefficients of six factors (family burden, individual resilience, family problem-solving and coping, inner family support, outer family social support, and family function) in the model were statistically significant (*P* < 0.05), indicating that all of them were significantly associated with family adaptation. Among them, inner family support, outer family social support, and family function were direct influencing factors, while the others were indirect influence factors. The path coefficients of the total effect of the determinants on family adaptation, from largest to smallest, were as follows: individual resilience (0.562), family function (0.483), outer family social support (0.345), family burden (−0.300), inner family support (0.293), problem-solving, and coping (0.127).

**Table 3:** Effects of factors and pathways associated with family adaptation.

**Factor**	**Standardized direct effects**		**Standardized indirect effects**			**Standardized total effects**
	**Pathways**	**Direct effect value**	**Pathways**	**Indirect effect value**	**Total indirect effect value**	
FB	–	–	FB—The pathway from IR to FA—FA	−0.300 = −0.533^*^0.562	−0.300	−0.300
IR	–	–	IR—FF—FA	0.223 = 0.461^*^0.483	0.562	0.562
			IR—The pathway from IFS to FA—FA	0.088 = 0.300^*^0.293		
			IR—The pathway from OFSS to FA—FA	0.160 = 0.463^*^0.345		
			IR—The pathway from PSC to FA—FA	0.091 = 0.127^*^0.717		
PSC	–	–	PSC—The pathway from OFSS to FA—FA	0.127 = 0.345^*^0.368	0.127	0.127
OFSS	OFSS—FA	0.202	OFSS—The pathway from IFS to FA—FA	0.143 = 0.488^*^0.293	0.143	0.345
IFS	IFS—FA	0.140	IFS—The pathway from FF to FA—FA	0.153 = 0.483^*^0.317	0.153	0.293
FF	FF—FA	0.483	–	–	–	0.483

* *This result means that an increase of one standard deviation of the outer family social support score led to an increase of 0.345 unit of family adaptation score.*

^*^FB, family burden; IR, individual resilience; OFSS, outer family social support; IFS, inner family support; PSC, problem-solving and coping; FF, family function; FA, family adaptation.

## Discussion

This study found that the average score of family adaptation was 76.00 (SD 18.35), and the average item score was 5.07, which was higher than the theoretical median (60 and 4). This score is consistent with the study by Wang involving patients with Alzheimer's disease (31). There is currently no cut-off value for this scale. Therefore, we cannot completely determine the level of family adaptation in patients with liver cancer. However, according to the rating method of the scale (from 1 = not at all to 7 = totally satisfied), four points mean that they have a neutral attitude toward the overall assessment of the family. It can be considered that the family members of the patients with liver cancer in this study were slightly satisfied with the overall adaptation of the family. The characteristics and structure of families of patients with liver cancer are vulnerable to the impact of cancer, which may lead to an adaptation crisis if effective measures are not taken in a sufficient period of time (43). According to the family system theory, the individual and system are intertwined and inseparable. The individual's quality of life is inevitably affected by the family's adaptation, and maladjustment of the family often affects the family members' quality of life (44). Therefore, exploring the influencing factors, paths, and effects of family adaptation is of practical significance and can provide a theoretical reference for family intervention programs for patients with liver cancer.

We developed a hypothetical model based on the McCubbin family resilience model to explain the factors that affect the family adaptation of patients with liver cancer, as well as tested the effectiveness of the model. In this model, family burden, individual resilience, and “problem-solving and coping” are indirect factors affecting family adaptation, while family function and support system directly affect family adaptation.

Family burden indirectly affects family adaptation through individual resilience, and the impact is relatively high. The studies by Ju et al. (45) and Tong (46) both showed that family burden is a significant risk factor for family resilience, but they did not explore the mediating role of individual resilience. However, the study by Hsiao and Van Riper (47) on families of children with Down syndrome found that when faced with a major stressful event, an individual's positive perception and significance of the family can help the family achieve a good state of adaptation. This finding is consistent with the accepted definition of individual resilience. A cancer diagnosis is a very stressful event for families with liver cancer, especially in China, where middle-aged men have a high incidence of liver cancer. These men are also the main source of income and spiritual support of the family. The diagnosis will then cause a serious burden on the family's economic status, daily activities, and entertainment (48). When facing serious negative stressful events, individuals will be affected first, which results in psychological and behavioral changes. Both Zhang et al. (49) and Wang et al. (50) studied the individual resilience of patients with cancer and found that family burden is an important factor affecting individual resilience, and a greater family burden correlated with lower individual resilience. Other studies have also confirmed a significant correlation between individual and family resilience. A study by Card and Barnett (51) showed that individual resilience plays an important role in family resilience, as it can help patients and family members actively evaluate and recognize stress, promote and develop family resilience, and family adaptability. Therefore, family burden can have an indirect effect on family adaptation by affecting individual resilience.

In this model, individual resilience had the highest impact on family adaptation. Although it has no direct effect on family adaptation, it plays a fundamental role in multiple influencing paths. Individual resilience can affect the perception of support systems, family problem-solving and coping, as well as family function by influencing individual psychological behavior, thus indirectly affecting family adaptation, which is similar to the findings of Kukihara et al. (52) and Han et al. (53). Resilience refers to an individual's ability to maintain and restore mental health in the face of stress or adversity (23). When facing major diseases, good individual resilience is an important family resilience factor. Higher levels of individual resilience indicate better family function, higher levels of perceived support, and better family problem-solving and coping abilities (26–28). However, it is generally believed that individual resilience is an intermediary factor between family resilience and other family outcomes, thus emphasizing the influence of family on individuals (54). However, as individual resilience is an essential factor affecting family resilience, people can explore more effective interventions to improve family resilience from this perspective (55, 56). The family system theory states that the individual and the family are intertwined. Being the functional unit of the family, the individual also plays a very important role in the process of family adjustment and adaptation (57). Meanwhile, when defining the concept of family adaptation, McCubbin considered it to be manifested as two levels of adaptation, that is, the “fitness” between individuals and the family as a whole, and between the family and its community or environment (20). To a certain extent, it also illustrates the importance of personal factors in promoting family adaptation. This also reminds clinical medical staff that in the field of family nursing practice for the care of cancer patients, researchers should not only regard the family as a whole, but also pay attention to the development and growth of individuals in the family, as well as clarify the role boundaries between the individual and family. This allows for the realization of two-way growth and well-rounded development of the individual and family, promoting family adaptation more effectively.

This study found that family problem-solving and coping indirectly affected family adaptation through outer family social support, with the lowest impact, which is consistent with the results of Mirsoleymani's study (58). This may be because higher levels of family problem-solving and coping abilities lead to greater ease for families of patients with liver cancer to perceive outside support, and thus, can actively use support resources to cope with pressure, thereby promoting family adaptation. In this study, the direct effects of “family problem-solving and coping” on inner family support, family function, and family adaptation were not significant, consistent with some previous studies' results (16, 38). Using the same scale with the families of older adults with dementia, Wang et al. (38) also reported a loss of direct effect of “family problem-solving and coping” on family resources and family adaptation, retaining only a direct effect on the outer family support system. This may be related to the limitations of the measurement scale itself. The scale used in this study is a revised Chinese version from an original foreign scale, which includes obtaining support, positive cognition, seeking support from spirit and belief, seeking social support (including support from other families, social institutions, doctors, and professionals), negative cognition, and avoidance. Most of them point to the cognition of family situation and search for support, which are highly correlated with outer family social support system. Meanwhile, traditional families often rely on their inner strength to solve and address various issues in Chinese cultural situations. Therefore, this scale may not be a good measure of Chinese family problem-solving and coping ability, which may lead to the final model retaining only one significant relationship between family problem-solving and coping with outer family social support. Following studies on family coping need to explore more localized and targeted measurement tools to further explore the family's problem-solving and coping skills when facing a crisis in family development and adaptation.

In this study, perceived support of liver cancer families was measured and divided into inner family support and outer family social support, both of which had direct and indirect effects on family adaptation. The role of support systems in family adjustment has been confirmed in many studies (31, 59, 60). The lack of inner family support networks and outer family social support can lead to family maladjustment. In particular, the less family support and social support people perceive, the easier it is for the family to have a low adaptation level, consistent with our research results. In this study, the direct effect of outer family social support on family function disappeared, and it affected family function and family adaptation through inner family support. This may be related to Chinese family culture. Many Chinese people believe that they have to solve their own family affairs. The inner family support system is the base of the outer family social support system, which in turn acts on the whole family through the former (61). Support from friends, communities, and society can provide families with informational and emotional assistance so that they can feel supported, thereby promoting communication and mutual support between family members and enhancing their perceived inner family support. A study by Mo'tamedi et al. (62) found that the inner family support system is an important factor in family resilience, and was positively correlated with family adaptation. This study also found that the overall effect of outer family social support on family adaptation was higher than that of inner family support. This may be because the impact of inner family support on family adaptation has a “ceiling effect.” In this study, the score of perceived inner family support was 22.06, with a full score of 28, and the score of perceived outer family social support was 39.56, with a full score of 56. The level of perceived inner family support is higher than that of outer family social support, which is consistent with the findings of Fontes et al. (63). Therefore, the changes in outer family social support may cause greater effects on family adaptation than inner family support.

Family function can directly affect family adaptation, with this effect being relatively high, consistent with the results of Mirsoleymani's study (58). The definitions of family function and family adaptation are not clear in the literature. Some studies use family functions to reflect family adaptation, which may lead to misunderstandings. In this study, the concepts of family function and family adaptation are different, with a distinction needed to be made. As an outcome indicator, family adaptation refers to the harmony and balance of the family; that is, the state of balance and stability achieved by the family through coping and efforts when facing a crisis (20). On the other hand, family function is used to describe the family's current internal characteristics and structure, which refers to the emotional connection between family members, family rules, family communication and the effectiveness of dealing with external events (64). First, good family function can provide a supportive environment for patients and their families, which can not only ensure that patients receive more physiological care and emotional support, but can also help regulate the psychological stress responses of family members. It can also help patients and family members establish good role adaptations so as to promote effective interaction among family members, which then helps the family achieve a good state of harmony and balance (52, 65). Therefore, researchers should focus on the important role of family function in liver cancer families in clinical nursing practice, explore more plans to strengthen family function, help families deal with various stressful events effectively, and finally achieve a balanced and stable state.

## Conclusions

Understanding the family adaptation to stressful events is central to promoting well-being in liver cancer families. In this study, family adaptation of liver cancer families was maintained at the level of mild satisfaction. It was affected by individual resilience, family function, support system, family problem-solving and coping ability. Therefore, in the practice of home care for liver cancer, clinical workers should pay not only special attention to direct influencing factors, adopt strategies to strengthen the overall family function, and encourage the active use of support systems, but also consider indirect influencing factors to improve patients' personal and family coping ability, reduce the burden on the family, and help the family maintain a harmonious and balanced state. Further research should explore the intervention strategies for the family adaptation of liver cancer patients, apply theories to practice, and continuously improve the care and services for liver cancer families.

## Limitations and Future Perspectives

Upon critically analyzing the present study, several limitations must be considered when interpreting our findings. First, self-report tools were used, which are not exempt from limitations such as inaccurate reporting. Second, participation in this study was voluntary, and some maladaptive families refused to participate and were not included in this study according to voluntary principles. Consequently, the study's sample composition may not represent the characteristics of all the liver cancer families in China. Third, the family-related variables in this study were reported by family caregivers and may not describe the family's overall situation comprehensively and accurately. Follow-up studies should further explore the difference between the outcomes reported by patients and those reported by family caregivers.

With the advancement of medical standards, the survival time of patients with liver cancer has been prolonged, and an increasing number of families have to coexist with such patients for a long time. Promoting better adaptation for families of patients with liver cancer has become an important issue. More research on family resilience is being carried out in China, and an increasing number of researchers are beginning to pay attention to family resilience and family adaptation of the diseased population (17, 18, 38, 42). In the future, we should continue to explore how to develop intervention programs that effectively promote family adaptation for patients with liver cancer based on the family resilience theory.

## Data Availability Statement

The raw data supporting the conclusions of this article will be made available by the authors, without undue reservation.

## Ethics Statement

The studies involving human participants were reviewed and approved by Ethics Committee of Zhongshan Hospital Affiliated to Fudan University. The patients/participants provided their written informed consent to participate in this study.

## Author Contributions

SM developed the study design, organized the sample recruitment, collected data, and contributed to the writing of the manuscript's introduction, discussion, and references sections. HL contributed to the study design and writing of the manuscript's introduction, discussion, and reference sections. YZ, JY, and XL assisted in the data collection and research design. JP contributed to the writing of the manuscript's introduction, discussion, and reference sections. YL contributed to the research design and literature review of this study. All authors contributed to the article and approved the submitted version.

## Funding

Funding was provided by the FuXing nursing research fund of Fudan University (FNF201906).

## Conflict of Interest

The authors declare that the research was conducted in the absence of any commercial or financial relationships that could be construed as a potential conflict of interest.

## Publisher's Note

All claims expressed in this article are solely those of the authors and do not necessarily represent those of their affiliated organizations, or those of the publisher, the editors and the reviewers. Any product that may be evaluated in this article, or claim that may be made by its manufacturer, is not guaranteed or endorsed by the publisher.
